# Supplementary material for: Combined dendritic cell and anti-TIGIT immunotherapy potentiates adaptive NK cells against HIV-1
Source: EMBO Mol Med. 2025 Jun 5;17(7):1756–93. doi: 10.1038/s44321-025-00255-x (PMC12254423; doi:10.1038/s44321-025-00255-x)
Supplement: Supplementary file 1 — Appendix [file 44321_2025_255_MOESM1_ESM.pdf]

Appendix

COMBINED DENDRITIC CELL AND ANTI-TIGIT IMMUNOTHERAPY POTENTIATES ADAPTIVE NK CELLS AGAINST HIV-1

Sánchez-Cerrillo et al.

Contents:

Appendix Figure S1 and legend. Phenotypical characterization of nano-PIC MDDC from PWH. Pages 2-3.

Appendix Figure S2 and legend. Analysis of cytotoxic function of NK cells primed with Nano-PIC MDDCs. Pages 4-5.

Appendix Figure S3 and legend. Levels of Checkpoint receptors in NK cells from different groups of PWH compared with HD and proportions of p24+ CD4+ T cells after TIGIT blocking antibody. Pages 6-8.

Appendix Figure S4 and legend. Characterization of adaptive NK cell subsets in humanized BLT mice during HIV-1 infection. Pages 9-10.

Appendix Figure S5 and legend. Analysis of memory NK cells subsets in humanized NSG mice transplanted with CD4+ T cells from PWH and combined anti-TIGIT and Nano-PIC/NK cell immunotherapy. Pages 11-12.

Appendix Table S1. Clinical and demographic parameters of the PWH selected cohort used to generate MDDC used for analysis of expression of cytokines, maturation markers and NK receptor ligands and in transwell experiments. Page 13.

Appendix Table S2. Clinical and demographic parameters of the PWH used for functional experiments and to analyze expression of exhaustion markers and TRAIL. Page 14.

Appendix Table S3. Intact HIV proviral DNA assay (IPDA) of intact and defective sequences detected in DNA from cultured CD4+ T cells from PWH. Page 15.

Appendix Table S4. Clinical and demographic parameters from the validation PWH cohort used for functional TIGIT and TRAIL blockade experiments. Page 16.

Appendix Table S5. Clinical and demographic parameters of the PWH used in the 3 mVOA experiments. Page 17.

Appendix Table S6. Exact p-values related to Figures 1-8. Pages 18-21.

Appendix Figure S1

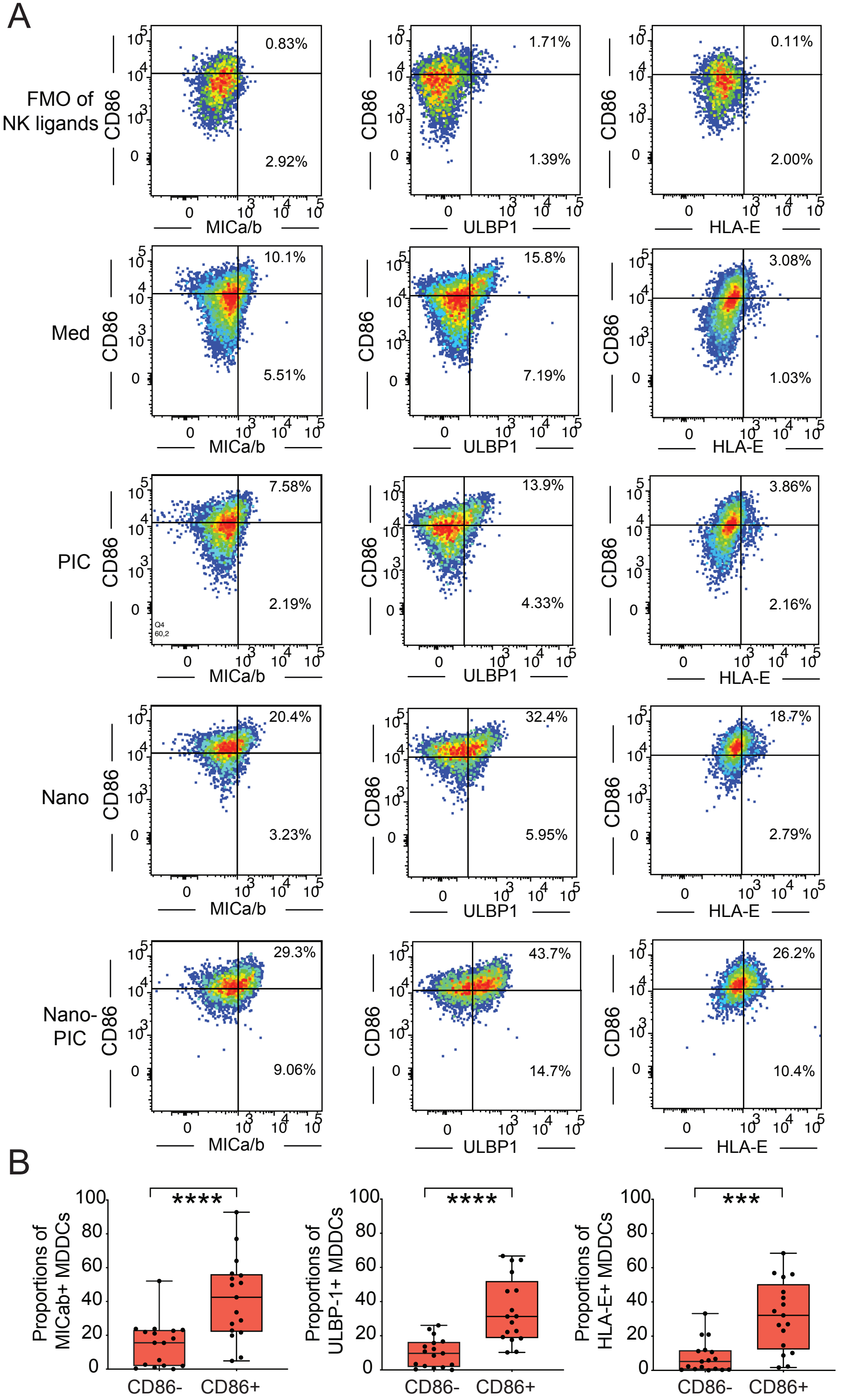

Appendix Figure S1. Phenotypical characterization of nano-PIC MDDC from PWH. (A): Representative flow cytometry dot plots showing expression ligands for NK receptors MICa/b, ULBP1 and HLA-E and surface levels of CD86 on MDDCs from a PWH donor after 16h culture in medium, sol PIC and with empty nano or Nano-PIC. Staining background control for NK ligands are included. (B): Proportions of MICa/b+ (left), ULBP1+ (middle) or HLA-E+ (right) in CD86- or CD86+ MDDC after 16h of stimulation with Nano-PIC in n=17 PWH donors. In panel (B) data are presented in Box and Whiskers plots showing median values and maximum and minimum error bars. Statistical significance was calculated using a two tailed Wilcoxon test.***$p<0.001$; ****$p<0.0001$.

Appendix Figure S2

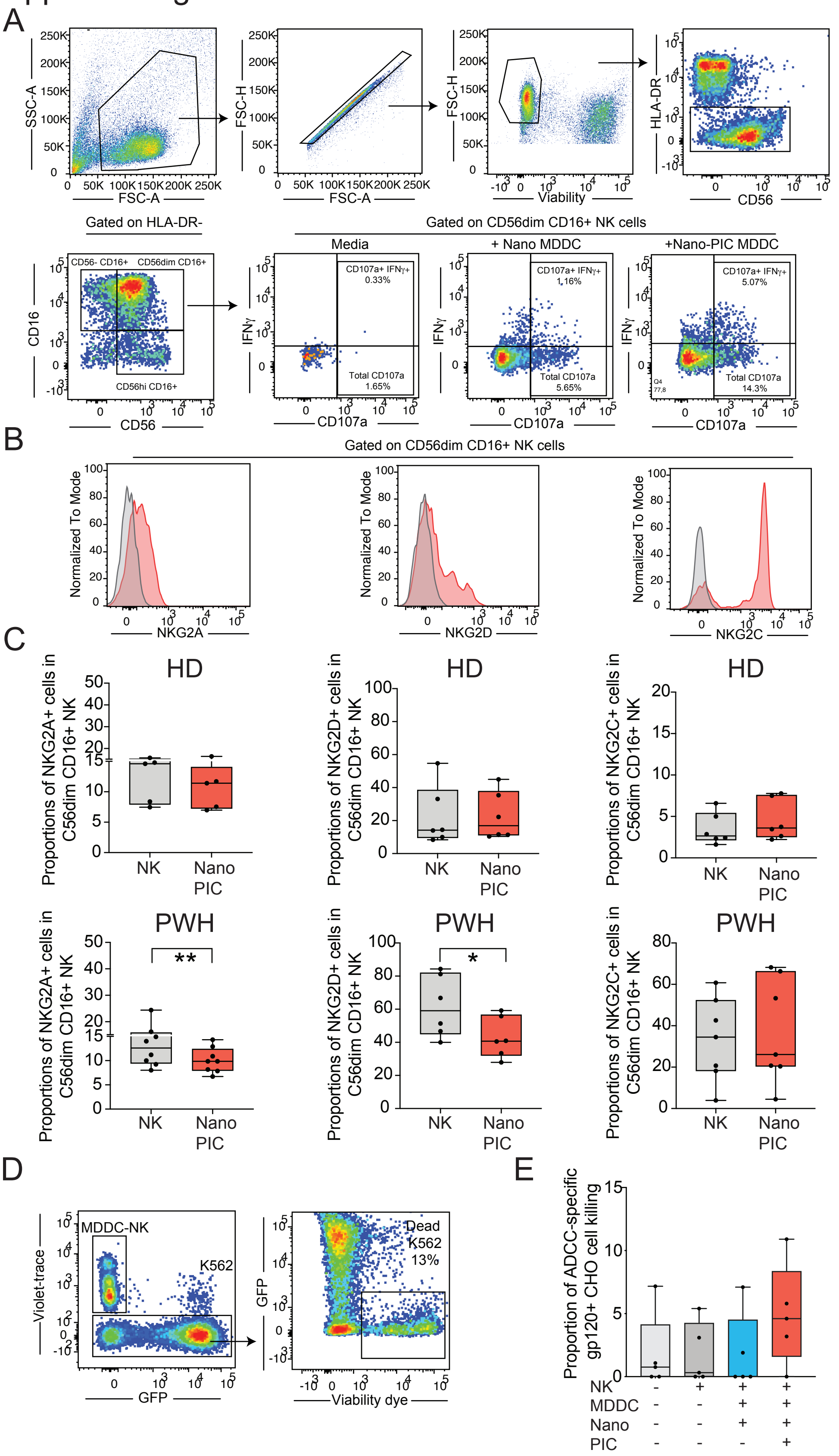

Appendix Figure S2. Analysis of cytotoxic function of NK cells primed with Nano-PIC MDDCs. (A): Representative flow cytometry gating strategy showing dot plots and defining NK cell subsets based on the expression of CD56 and CD16 and intracellular expression of IFNg and CD107a in NKs cultured in the absence (49000 events acquired) or in the presence of MDDC activated with empty nano (88000 events acquired) or Nano-PIC (78000 events acquired) from a PWH example in CD56dim CD16+ NK without another stimulus is represented. (B, C): Representative flow cytometry of NKG2A (left), NKG2D (middle) and NKG2C (right) histograms from CD56dim CD16+ NK from PWH. FMO is included for each marker in gray (B). Analysis of proportions of NKG2A (left panel), NKG2D (middle panel) and NKG2C (right panel) on CD56dim CD16+ NK cells cultured in the absence or the presence of MDDC stimulated with PIC-loaded nanoparticles in the same culture from HD (n=6) and PWH (n=6) (C). (D): Flow cytometry gating strategy used to analyze NK mediated killing of target K562-GFP cell line in the presence of violet cell trace-labelled MDDC and NK cells. Dead target K562 cells were considered as violet- cells losing GFP and gaining cell death viability dye expression. (E): Quantification of antibody-dependent cellular cytotoxicity (ADCC) killing of CHO target cells overexpressing HIV-1 gp120 protein in the presence of a cocktail of HIV-1 specific bNAbs (VCR01; PGT121; 3BNC117) and NK cells individually or stimulated with Nano or Nano-PIC DC. Values were subtracted from baseline levels. In panel C and D data are presented in Box and Whiskers plots showing median values and maximum and minimum error bars. Statistical significance was calculated using a two tailed Wilcoxon test. *$p<0.05$; **$p<0.01$.

Appendix Figure S3

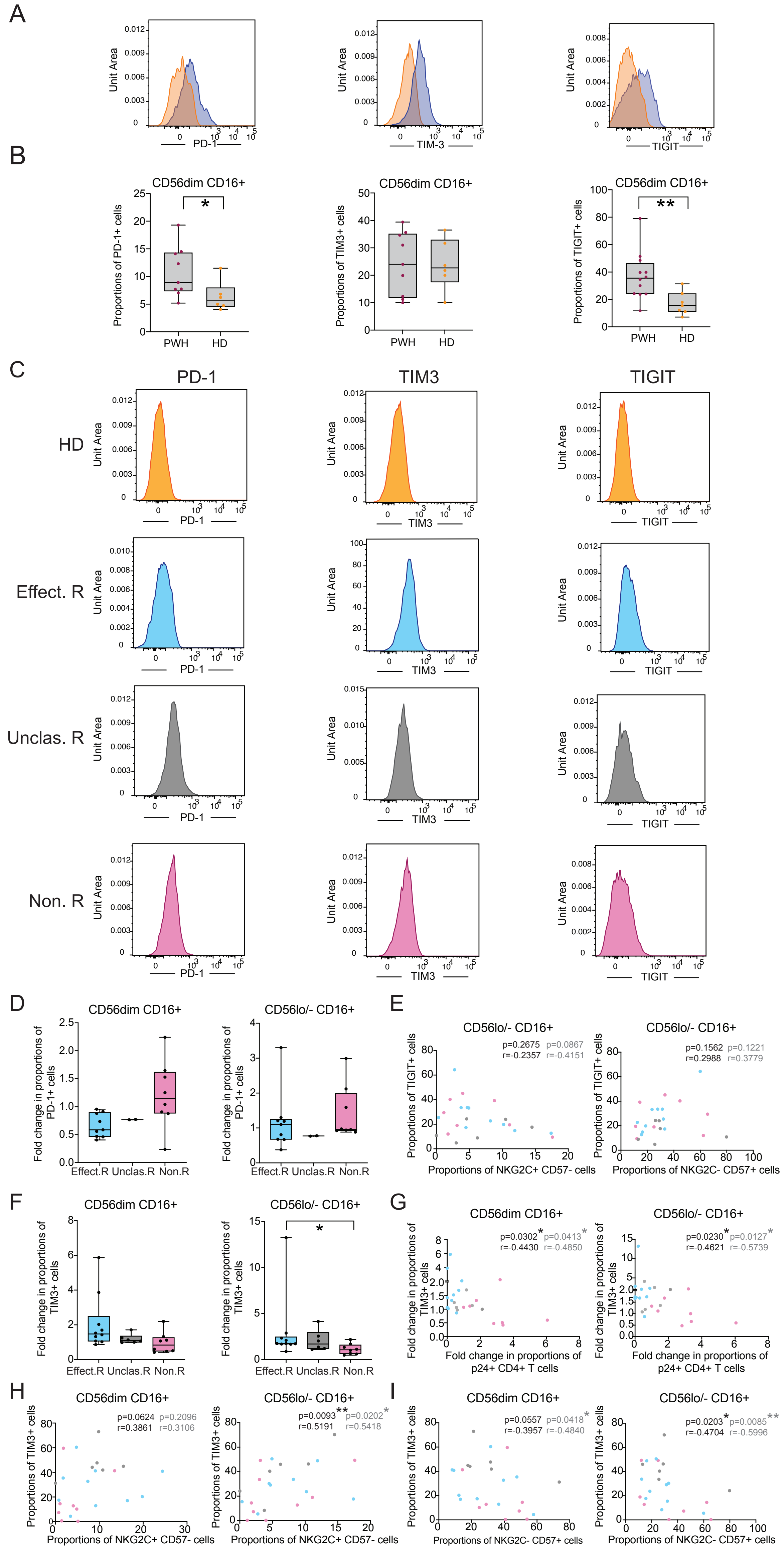

Appendix Figure S3. Levels of Checkpoint receptors in NK cells from different groups of PWH compared with HD and proportions of p24+ CD4+ T cells after TIGIT blocking antibody. (A): Representative flow cytometry histograms showing expression of PD-1 TIM3 and TIGIT in gated CD56dim CD16+ NK cells from a representative PWH after culture with Nano-PIC MDDC. Background levels of FMO are overlayed in orange. (B): Baseline levels of PD-1+, TIM3+ and TIGIT+ cells in CD56dim CD16+ NK cells from n=9 PWH compared with n=6 HD. (C): Representative flow cytometry histograms showing baseline expression of PD-1 TIM3 and TIGIT in gated CD56dim CD16+ NK cells from representative HD, effective responder (Effect-R), unclassified (Unclas.R) and non-responder (Non-R) PWH (D): Fold change in PD-1+ cells compared to baseline levels present in NK included in CD56dim (left) or CD56lo/- (right) CD16+ subsets from effective responder (n=10; Effect. R; blue), unclassified (n=6; Unclas. R; gray) and non-responder (n=8; Non-R.; pink) PWH after Nano-PIC MDDC treatment. (E): Spearman correlations between proportions of TIGIT+ cells and proportions of NKG2C+ CD57- adaptive NK subset (left) and NKG2C- CD57+ effector subset (right) after Nano-PIC MDDC within CD56lo/- CD16+ NK. (F): Fold change in TM3+ cells in the same conditions previously described in (D). (G, H, I): Spearman correlations between fold change proportions of TIM3 within CD56dim (left) or CD56lo/- (right) CD16+ NK and fold change proportions of p24+ CD4+ T cells after Nano-PIC MDDC. Spearman correlations between proportions of TIM3 and proportions of NKG2C+ CD57- adaptive NK subset (H) and NKG2C- CD57+ effector subset (I) after Nano-PIC MDDC within CD56dim (left) and CD56lo/- (right) CD16+ NK. Spearman statistical P and R values considering all data (black) and without unclassified group (gray) are shown. In C-I panels, PWH were highlighted in blue, gray and pink to define effective, unclassified, and dysfunctional responder groups, respectively. In panel B, D and F data are presented in Box and Whiskers plots showing median values and maximum

and minimum error bars. Statistically significant differences were calculated using a spearman test and a Kruskal-Wallis test for multiple comparisons. *$p<0.05$; **$p<0.01$.

Appendix Figure S4

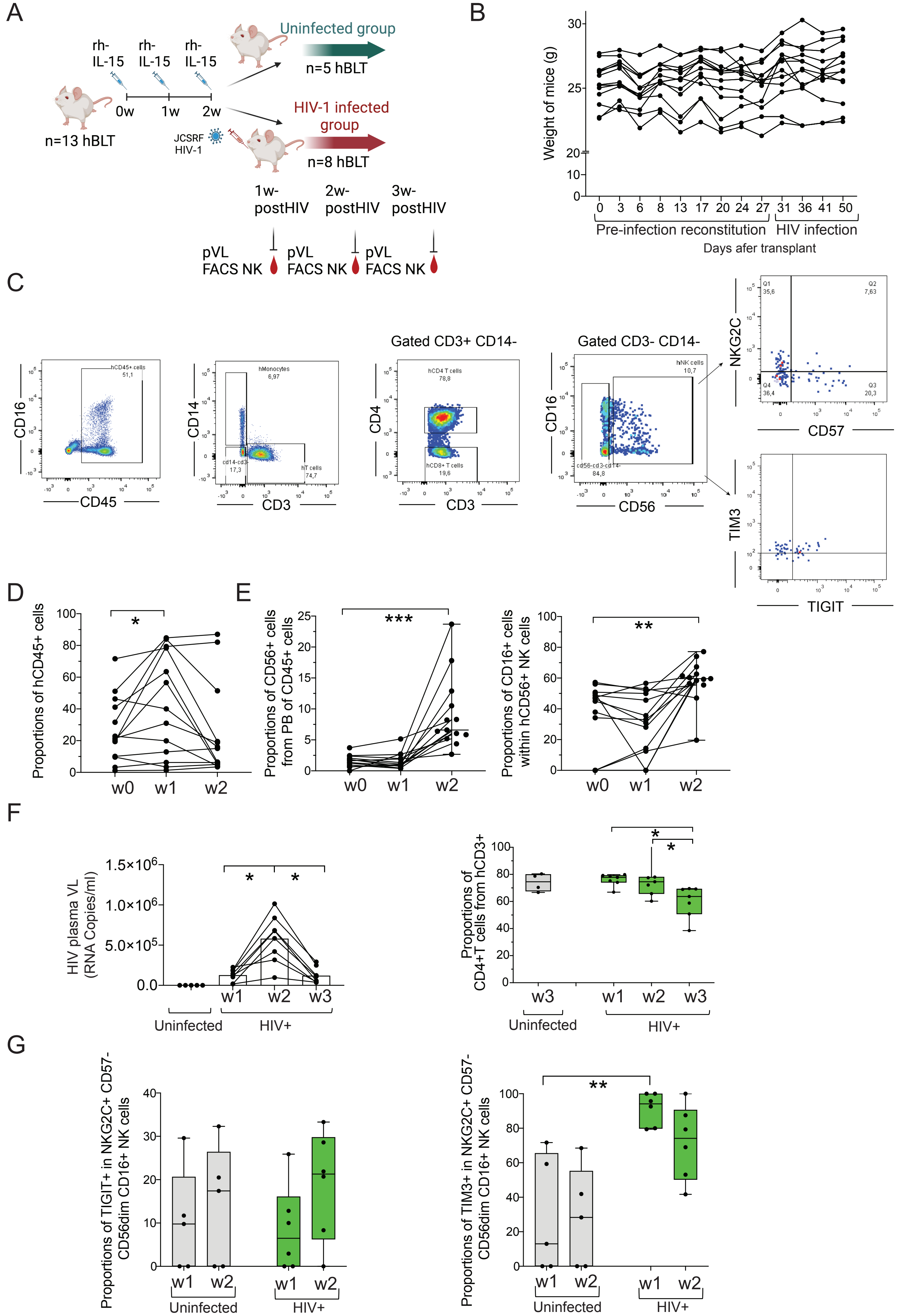

Appendix Figure S4. Characterization of adaptive NK cell subsets in humanized BLT mice during HIV-1 infection. (A): Schematic of flow experiment representing times of reconstitution of human immune cells and infection points. (B): Weigh (g) of individual hBLT mice used for the in vivo experiment before and after HIV-1 infection. (C): Flow cytometry gating strategy from peripheral blood of a representative hBLT mouse identifying hCD45+ cells and discriminating between myeloid CD14+ versus CD3+ T cells. CD4+ T cells are identified within CD3+ cells and NK cells are defined by CD56 vs CD16 expression in CD14- CD3- cells. Expression of NKG2C, CD57, TIM3 and TIGIT on gated NK is also shown (D, E): Proportions of human CD45+ (D), total and CD16+ CD56dim NK cells within the pool of human lymphocytes (E) in the peripheral blood of BLT mice before (Wk0) and after 1 and 2 weeks of injection with recombinant human IL-15. (F): HIV-1 plasma viral load (copies HIV-1 RNA/mL; left) and proportions of CD4+ T cells within CD3+ T cells (right) in uninfected and HIV-1 infected BLT mice at 1, 2, 3 weeks post-infection. (G): Analysis of proportions of TIGIT+ (left) and TIM3+ (right) in adaptive NKG2C+ CD57- precursors in uninfected (grey) or HIV infected (green) hBLT mice at 1 and 2 weeks. In panel (F-G) data are presented in Box and Whiskers plots showing median values and maximum and minimum error bars. Statistical significant differences between different mouse groups or in the same animals over time were calculated using two-tailed Mann Whitney and Wilcoxon pair matched tests, respectively and Bonferroni correction for multiple comparisons. *p<0.05; **p<0.01; ***p<0.001.

Appendix Figure S5

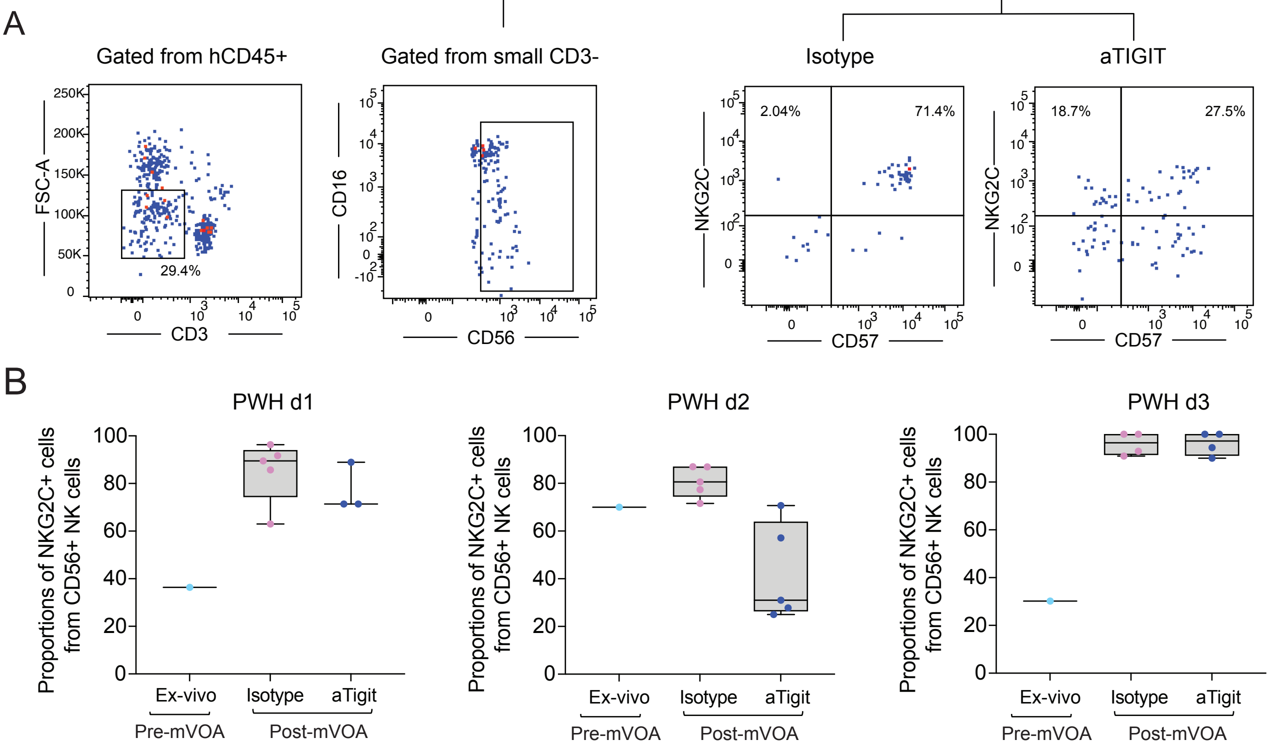

Appendix Figure S5. Analysis of memory NK cells subsets in humanized NSG mice transplanted with CD4+ T cells from PWH and combined anti-TIGIT and Nano-PIC/NK cell immunotherapy. (A): Representative flow cytometry gating strategy showing the identification of human NK cells from CD3- human lymphocytes based on expression of CD56 and CD16 and expression of NKG2C and CD57 on gated NK cell. Combined FACS data from n=14 isotype and n=14 anti-TIGIT treated NSG mice are shown to maximize number of events for gated NK and facilitate detection of subpopulations. (B): Proportions of total NKG2C+ adaptive NK cells for each PWH donor d1, d2, d3 used in the three independent *in vivo* experiments at pre-mVOA in after mVOA timepoints. Data from panel B are presented in Box and Whiskers plots showing median values and maximum and minimum error bars.

	PWH
**PWH n**	21
**CD4 T cell count median (min-max). n.d (n/N)**	832 (466-2406)<br>1 (20/21)
**CD4 T cells NADIR count median (min-max). n.d (n/N)**	363 (19-869)<br>6 (15/21)
**Ratio CD4/CD8+ T cells count median (min-max). n.d (n/N)**	0.915 (0.22-2.14)<br>1 (20/21)
**Age (years) median (min-max). n.d (n/N)**	54 (25-74)<br>1 (20/21)
**Time on ART (Years) median (min-max). n.d (n/N)**	12 (2-28)<br>1 (20/21)
**Sex (% male)**	95
**Viral load at sample recruitment (RNA copies/ml).**	<20
**Viral load at diagnosis (RNA copies/ml). n.d (n/N)**	54650 (99-5940000)<br>9 (12/21)
**Co-infection with HCV**	No
**CMV (% IgG positive). n.d (n/N)**	100<br>6 (15/21)

Appendix Table S1. Clinical and demographic parameters of the PWH selected cohort used to generate MDDC used for analysis of expression of cytokines, maturation markers and NK receptor ligands and in transwell experiments.

Abbreviations: n.d: no date.

	Total PWH	Effective-responders	Unclassified-responders	Non-responders	P values (Effect. R vs Non-R.)
**PWH n**	33	13	7	13	
**CD4 T cell count median (min-max). n.d (n/N)**	835 (432-1639)<br>1 (32/33)	1099 (626-1639)	771 (432-1586)	829 (596-1160)<br>1 (12/13)	0.09
**CD4 T cells NADIR count median (min-max). n.d (n/N)**	413 (9-869)<br>6 (27/33)	457 (150-596)<br>2 (11/13)	261.5 (162-502)<br>1 (6/7)	362 (9-869)<br>3 (10/13)	0.32
**Ratio CD4/CD8+ T cells count median (min-max). n.d (n/N)**	0.995 (0.37-2.44)<br>1 (32/33)	1.1 (0.51-2.44)	0.93 (0.37-2.17)	0.88 (0.4-1.68)<br>1 (12/13)	0.01*
**Age (years) median (min-max). n.d (n/N)**	53 (25-77)<br>1 (32/33)	45 (25-71)	57 (31-77)	56 (37-75)<br>1 (12/13)	0.03*
**Time on ART (Years) median (min-max). n.d (n/N)**	9.5 (1.5-27)<br>2 (31/33)	8.5 (2-25)<br>1 (12/13)	9.5 (3-27)	13 (1.5 -24)<br>1 (12/13)	0.59
**Sex (% male)**	97	100	100	92.3	0.82
**Viral load at sample recruitment (RNA copies/ml).**	<20	<20	<20	<20	
**Viral load at diagnosis (RNA copies/ml). n.d (n/N)**	100000 (3810-2600000)<br>12 (21/33)	125000 (3810-2600000)<br>5 (8/13)	125000 (16-256000)<br>2 (5/7)	76500 (99-825000)<br>5 (8/13)	0.57
**Co-infection with HCV**	No	No	No	No	
**CMV (% IgG positive). n.d (n/N)**	69.7<br>10 (23/33)	82<br>2 (11/13)	60<br>2 (5/7)	71<br>6 (7/13)	0.06

Appendix Table S2. Clinical and demographic parameters of the PWH used for functional experiments and to analyze expression of exhaustion markers and TRAIL.
Abbreviations: n.d: no date.

	Conditions	HIV-DNA regions	Copies per million CD4 +T cells
**ART 121**	CD4	Hypermutated and/or 3' deletion	17,31187017
		5' deletion	103,8763352
		Intact	98,18737393
	CD4+NK	Hypermutated and/or 3' deletion	16,37502322
		5' deletion	32,75026184
		Intact	190,2797848
	CD4+NK+Nano-PIC MDDC	Hypermutated and/or 3' deletion	121,6973663
		5' deletion	162,2642187
		Intact	595,000424
**ART 126**	CD4	Hypermutated and/or 3' deletion	361,1802458
		5' deletion	444,5689774
		Intact	56,34632819
	CD4+NK	Hypermutated and/or 3' deletion	449,3259858
		5' deletion	565,1250136
		Intact	19,58875044
	CD4+NK+Nano-PIC MDDC	Hypermutated and/or 3' deletion	832,2548146)
		5' deletion	770,575719
		Intact	60,65527812
**ART 129**	CD4	Hypermutated and/or 3' deletion	1570,651029
		5' deletion	1534,939289
		Intact	505,4230736
	CD4+NK	Hypermutated and/or 3' deletion	1631,824781
		5' deletion	1059,779424
		Intact	200,6887376
	CD4+NK+Nano-PIC MDDC	Hypermutated and/or 3' deletion	2765,063755
		5' deletion	2325,216705
		Intact	170,3128094
**ART 127**	CD4	Hypermutated and/or 3' deletion	202,4481764
		5' deletion	224,9468923
		Intact	80,2103115
	CD4+NK	Hypermutated and/or 3' deletion	328,3944469
		5' deletion	451,5690127
		Intact	90,7199522
	CD4+NK+Nano-PIC MDDC	Hypermutated and/or 3' deletion	418,382786
		5' deletion	481,1592751
		Intact	27,7938166

Appendix Table S3. Intact HIV proviral DNA assay (IPDA) of intact and defective sequences detected in DNA from cultured CD4+ T cells from PWH.
Abbreviations: Nano: nanoparticle; PIC: poly I:C; NK: natural killer.

	Total PWH	Effective responders	Non-responders	P values (Effect. R vs Non-R.)
**PWH n**	20	11	9	
**CD4 T cell count median (min-max). n.d (n/N)**	947 (432-2378)	992,5 (432-2378)	957 (643-1676)	0.95
**CD4 T cells NADIR count median (min-max). n.d (n/N)**	311 (11-504)<br>3 (17/20)	304 (11-504)<br>1 (10/11)	381,9 (245-502)<br>2 (7/9)	0.33
**Ratio CD4/CD8+ T cells count median (min-max). n.d (n/N)**	1.07 (0.37-2.54)	1.165 (0.37-1.49)	1.03 (0.65-2.54)	0.53
**Age (years) median (min-max). n.d (n/N)**	44,5 (25-74)	44,5 (25-57)	53 (31-74)	0.16
**Time on ART (Years) median (min-max). n.d (n/N)**	10.5 (4-25)<br>1 (19/20)	11.5 (4-25)	10.25 (5.5 -25)<br>1 (8/9)	0.64
**Sex (% male)**	100	100	100	>0.99
**Viral load at sample recruitment (RNA copies/ml).**	<20	<20	<20	
**Viral load at diagnosis (RNA copies/ml). n.d (n/N)**	136500 (2220-769000)<br>3 (17/20)	207500 (4500-769000)	67000 (2220- 528000)<br>3 (6/9)	0.29
**Co-infection with HCV**	No	No	No	
**CMV (% IgG positive). n.d (n/N)**	77,7<br>1 (19/20)	80	71,4<br>1 (8/9)	0.13

Appendix Table S4. Clinical and demographic parameters from the validation PWH cohort used for functional TIGIT and TRAIL blockade experiments.
Abbreviations: n.d: no date.

	Donor exp 1	Donor exp 2	Donor exp 3
**CD4 T cell count**	1148	989	650
**CD4 T cells NADIR count**	420	404	172
**Ratio CD4/CD8+ T cells count**	0.78	1.2	1.1
**Age (years)**	62	40	48
**Time on ART (Years)**	16	9	9
**Sex**	Male	Male	Male
**Viral load at diagnosis (RNA copies/ml)**	53000	10063	183000
**CMV IgG**	Positive	Positive	Positive
**NKG2C (%)**	36.4	70	30.2

Appendix Table S5. Clinical and demographic parameters of the PWH used in the 3 mVOA experiments. Experiment (Exp)

Appendix Table S6. Exact p-values related to Figures 1-8.

FIGURE 1B	Conditions	p-value
HD	Med vs nano-PIC	*p=0.0312
	Sol-PIC vs nano-PIC	*p=0.0312
	Nano vs nano-PIC	*p=0.0312
PWH	Med vs nano-PIC	*p=0.0156
	Sol-PIC vs nano-PIC	*p=0.0156
	Nano vs nano-PIC	*p=0.0312
**FIGURE 1C**	**Conditions**	**p-value**
HD	Med vs sol-PIC	*p=0.0268
	Med vs nano-PIC	***p=0.0004
	Nano vs nano-PIC	**p=0.0036
PWH	Med vs sol-PIC	*p=0.0136
	Med vs nano	**p=0.001
	Med vs nano-PIC	**p=0.002
	Sol-PIC vs nano-PIC	**p=0.0068
**FIGURE 1D**	**Conditions**	**p-value**
HD MICa/b	Med vs nano-PIC	***p=0.0006
	Sol-PIC vs nano-PIC	***p=0.0006
	Nano vs nano-PIC	***p=0.0006
HD ULBP-1	Med vs nano-PIC	***p=0.0006
	Sol-PIC vs nano-PIC	***p=0.0006
	Nano vs nano-PIC	*p=0.0312
HD HLA-E	Med vs nano-PIC	***p=0.0006
	Sol-PIC vs nano-PIC	**p=0.009
	Nano vs nano-PIC	***p=0.0006
PWH MICa/b	Med vs sol-PIC	**p=0.0072
	Med vs nano	**p=0.0042
	Med vs nano-PIC	**p=0.0012
	Sol-PIC vs nano	***p=0.0006
	Sol-PIC vs nano-PIC	**p=0.0012
	Nano vs nano-PIC	*p=0.02
PWH ULBP-1	Med vs sol-PIC	*p=0.01
	Med vs nano	**p=0.0012
	Med vs nano-PIC	**p=0.0012
	Sol-PIC vs nano	**p=0.0012
	Sol-PIC vs nano-PIC	**p=0.0012

	Nano vs nano-PIC	*p=0.0366
PWH HLA-E	Med vs nano	**p=0.0012
	Med vs nano-PIC	**p=0.0012
	Sol-PIC vs nano	**p=0.003
	Sol-PIC vs nano-PIC	**p=0.0012
	Nano vs nano-PIC	*p=0.0486
**FIGURE 2A**	**Conditions**	**p-value**
HD total CD107a+ cells	NK vs NK+ nano-PIC MDDC	*p=0.011
	NK + nano MDDC vs NK+ nano-PIC MDDC	*p=0.0417
HD CD107a+ IFNγ+ cells	NK vs NK+ nano-PIC MDDC	*p=0.011
PWH total CD107a+ cells	NK vs NK+ nano MDDC	*p=0.0219
	NK + nano MDDC vs NK+ nano-PIC MDDC	***p=0.0002
PWH CD107a+ IFNγ+ cells	NK vs NK+ nano-PIC MDDC	****p<0.0001
**FIGURE 2B**	**Conditions**	**p-value**
HD	Nano contact vs nano-PIC contact	*p=0.0406
	Nano-PIC contact vs nano-PIC non-contact	*p=0.0406
PWH	NK vs nano-PIC contact	**p=0.0053
	Nano-PIC contact vs nano-PIC non-contact	**p=0.0098
**FIGURE 2C**	**Conditions**	**p-value**
HD	NK vs nano-PIC MDDC	**p=0.0029
	NK+ Nano MDDC vs NK+ nano-PIC MDDC	*p=0.0167
PWH	NK vs nano-PIC MDDC	**p=0.0014
	NK+ Nano MDDC vs NK+ nano-PIC MDDC	*p=0.0373
**FIGURE 3G**	**Conditions**	**p-value**
Effect. R	CD4 Ral+RMD vs CD4+NK+Nano-PIC MDDC	**p=0.0015
Non-R.	CD4 Ral+RMD vs CD4+NK+Nano-PIC MDDC	*p=0.0156
	CD4+NK vs CD4+NK+Nano-PIC MDDC	*p=0.0356
**FIGURE 3H**	**Conditions**	**p-value**
CD56dim CD16+ NK cells	Effect. R vs Non-R	****p<0.0001
CD56lo/- CD16+ NK	Effect. R vs Non-R	***p=0.0005
**FIGURE 3I**	**Conditions**	**p-value**

CD56dim CD16+ NK cells	Effect. R vs Non-R	*p=0.0142
CD56lo/- CD16+ NK	Effect. R vs Non-R	*p=0.0144
**FIGURE 3J**	**Conditions**	**p-value**
CD56dim CD16+ NK cells	Effect. R vs Non-R	**p=0.0023
CD56lo/- CD16+ NK	Effect. R vs Non-R	**p=0.0041
**FIGURE 3K**	**Conditions**	**p-value**
Proportions of CD107a+ IFNγ+ NK cells	NKG2C+ CD57- vs NKG2C- CD57+	**p=0.0016
Proportions of CD107a+ GZB+ NK cells	NKG2C+ CD57- vs NKG2C- CD57+	**p=0.001
	NKG2C+ CD57+ vs NKG2C- CD57+	*p=0.0417
**FIGURE 4A**	**Conditions**	**p-value**
FC in proportions of TIGIT in CD56dim CD16+	Effect. R vs Unclas. R	*p=0.0326
	Effect. R vs Non-R	*p=0.0234
**FIGURE 4D**	**Conditions**	**p-value**
All donors	CD4 vs aTIGIT	**p=0.0036
	Isotype vs aTIGIT	***p=0.0008
Non-R.	CD4 vs Isotype	**p=0.0085
	Isotype vs aTIGIT	***p=0.0002
**FIGURE 5A**	**Conditions**	**p-value**
Proportions of MICa/b	p24- vs p24+	*p=0.0156
Proportions of ULBP-1	p24- vs p24+	*p=0.0156
Proportions of HLA-E	p24- vs p24+	*p=0.0156
**FIGURE 5B**	**Conditions**	**p-value**
FC in proportions of TRAIL in NKG2C+ CD57-	NK vs NK+ nano-PIC MDDC	*p=0.0177
FC in proportions of TRAIL in NKG2C-	NK vs NK+ nano-PIC MDDC	*p=0.0177
**FIGURE 5C**	**Conditions**	**p-value**
Proportions of TRAIL in CD56lo/- CD16+ NK	NKG2C+ CD57+ vs NKG2C+ CD57- (Effect. R)	*p=0.0294
	NKG2C+ CD57+ (Effect. R) vs NKG2C+ CD57+ (Non-R)	**p=0.0087
	NKG2C+ CD57+ (Unclas. R) vs NKG2C+ CD57+ (Non-R)	*p=0.0141
**FIGURE 5E**	**Conditions**	**p-value**
Proportions of TRAIL in NKG2C+ CD57+	Isotype vs aTIGIT	**p=0.0078
**FIGURE 5F**	**Conditions**	**p-value**
Ratio TIGIT/TRAIL+ cells in NKG2C- CD57+ CD56dim CD16+ NK	Unclas. R vs non-R	*p=0.02
Ratio TIGIT/TRAIL+ cells in NKG2C- CD57+ CD56lo/- CD16+ NK	Effect. R vs non-R	*p=0.0342
	Unclas. R vs non-R	*p=0.0426
**FIGURE 5G**	**Conditions**	**p-value**

FC in proportions of p24+ CD4+ T cells	CD4 vs Isotype	*p=0.0128
	Isotype vs aTRAIL	***p=0.0007
**FIGURE 6B**	**Conditions**	**p-value**
Proportions of NKG2C+ CD57+	Uninfected w2 vs HIV+ w2	*p=0.0346
**FIGURE 6D**	**Conditions**	**p-value**
Ratio TIM3/TIGIT in NKG2C+ CD57-	Uninfected w1 vs HIV+ w1	*p=0.0174
**FIGURE 7B**	**Conditions**	**p-value**
Proportions of p24+ cells	CD4 vs aTIGIT	*p=0.0255
**FIGURE 7C**	**Conditions**	**p-value**
Proportions of p24+ clusters >800um	CD4 vs aTIGIT	*p=0.0306
	Isotype vs aTIGIT	***p=0.0009
**FIGURE 7D**	**Conditions**	**p-value**
Number of GZB+ p24-/total area	p24 low zone vs p24hi zone	**p=0.0066
**FIGURE 8A**	**Conditions**	**p-value**
Proportions of NKG2C+ CD57- vs NKG2C+ CD57+	Pre-mVOA vs aTIGIT	**p=0.0029
	Isotype vs aTIGIT	**p=0.0013
Proportions of NKG2C+ CD57+ in CD56+ NK	Isotype vs aTIGIT	**p=0.0035
**FIGURE 8C**	**Conditions**	**p-value**
Proportions of TRAIL+ cells from total NKG2C	Isotype vs aTIGIT	*p=0.0221
