# Supplementary figures and images for: Combined dendritic cell and anti-TIGIT immunotherapy potentiates adaptive NK cells against HIV-1
Source: EMBO Mol Med. 2025 Jun 5;17(7):1756–93. doi: 10.1038/s44321-025-00255-x (PMC12254423; doi:10.1038/s44321-025-00255-x)

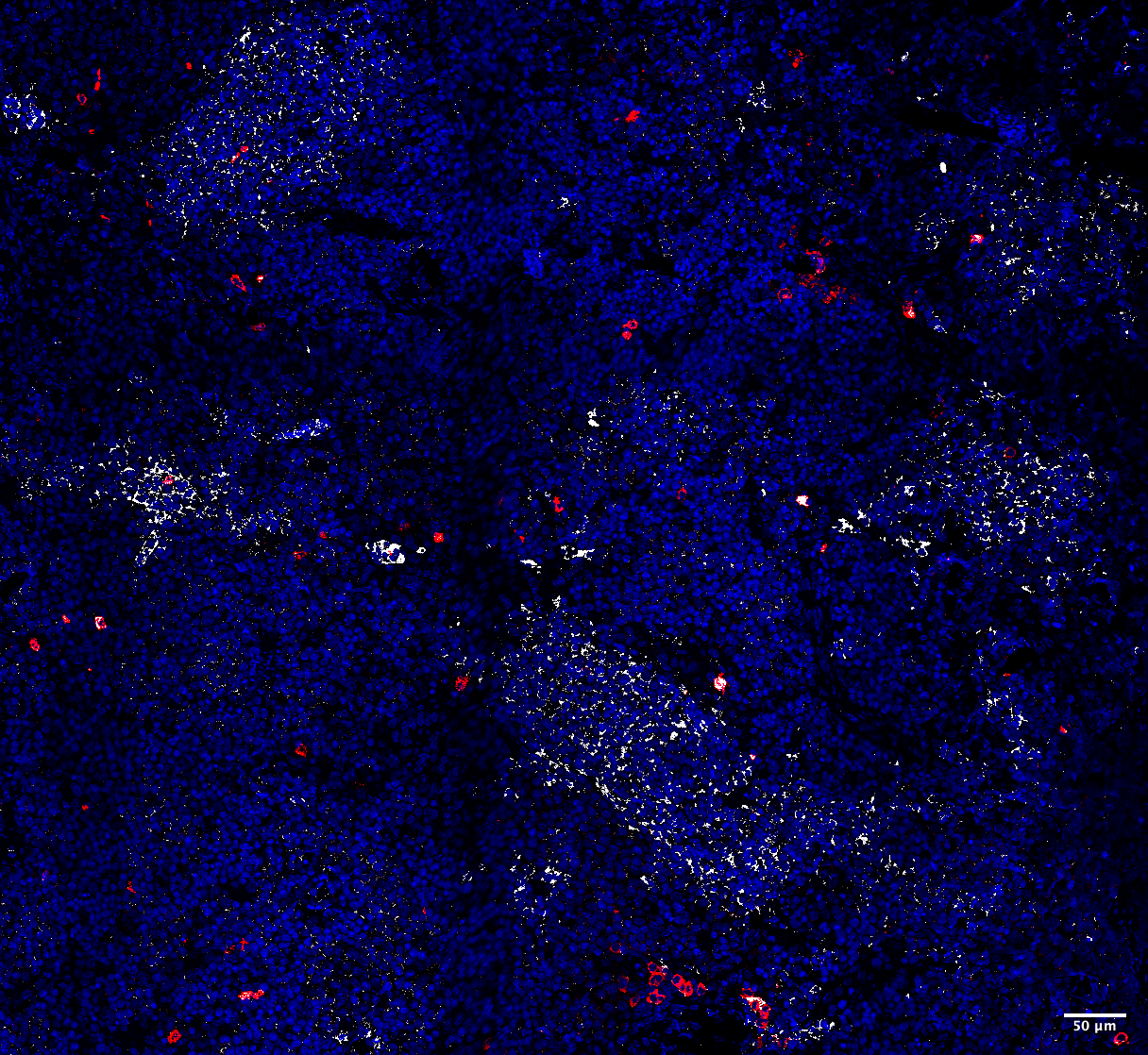

Supplement: Supplementary file 9 — Source data Fig. 7 [file 44321_2025_255_MOESM9_ESM.zip › Figure 7/7C/P24 Granzyme B ART62 SP13.tif]

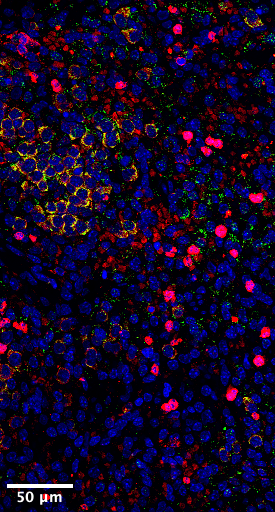

Supplement: Supplementary file 10 — Source data Fig. 8 [file 44321_2025_255_MOESM10_ESM.zip › Figure 8/8B/ART33 SP12 NKG2C AND TRAIL MERGE.tif]

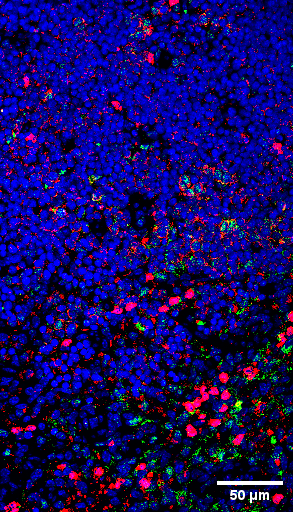

Supplement: Supplementary file 10 — Source data Fig. 8 [file 44321_2025_255_MOESM10_ESM.zip › Figure 8/8B/ART33 SP7 NKG2C TRAIL MERGE.tif]
