# Supplementary material for: Combined dendritic cell and anti-TIGIT immunotherapy potentiates adaptive NK cells against HIV-1
Source: EMBO Mol Med. 2025 Jun 5;17(7):1756–93. doi: 10.1038/s44321-025-00255-x (PMC12254423; doi:10.1038/s44321-025-00255-x)
Supplement: Supplementary file 11 — Expanded View Figures [file 44321_2025_255_MOESM11_ESM.pdf]

Expanded View Figures

Figure EV1. Phenotypical analysis of MDDC from healthy donors and PWH in presence of soluble or nanoparticle-encapsulated Poly I:C.

(**A**) Representative flow cytometry gating strategy defining viable big size HLA-DR + , CD14lo/-, CD11c+ and CD1a+ MDDC generated from Mo in the presence of GM-CSF and IL-4. (**B**) Proportions of live viability dye negative MDDCs from $n = 8$ HD (left panel) and $n = 8$ PWH (right panel). (**C**) Representative flow cytometry dot plots showing expression of CD86 vs intracellular staining of IFNβ in MDDCs from PWH cultured in media and after soluble (Sol PIC) or Nanoparticle (Nano PIC) -Poly I:C treatments. FMO for IFNβ is shown (left). Analysis of proportions of MDDCs from HD ($n = 8$; left) and PWH ($n = 9$; right) expressing intracellular IFNβ after 6 h of stimulation with soluble (Sol) or nanoparticle encapsulated poly: IC (Nano-PIC). (**D**) Analysis of IL-2, IL-12, IL-6, Il-15, IFNβ, IFNγ and TNFα, concentrations (pg/ml) present in culture supernatants of MDDCs from HD (left) an PWH (right) at 16 h in the mentioned conditions. (**E**) Basal mean fluorescence intensity (MFI) of CD86 (left) and CD40 (right) on MDDCs from $n = 15$ or $n = 8$ HD and $n = 13$ or $n = 7$ PWH, respectively. (**F–H**): MFI levels of CD86 (**F**) on MDDCs from $n = 15$ HD (left panel) and $n = 17$ PWH (right panel) at 6 h (**F**) or CD40 after 6 (**G**) and 16 h (**H**) of culture from $n = 7$ and $n = 7$ HD or $n = 8$ and $n = 10$ PWH, respectively, in basal conditions and after Sol PIC or nano empty or loaded with PIC treatments. Data in (**B–H**) are presented in Box and Whiskers plots showing median values and maximum and minimum error bars. Statistical significance between experimental conditions in the same samples were calculated using a two-tailed Wilcoxon tests and Bonferroni correction for multiple comparisons. $^{*}P < 0.05$; $^{**}P < 0.01$; $^{***}P < 0.001$.

© The Author(s)

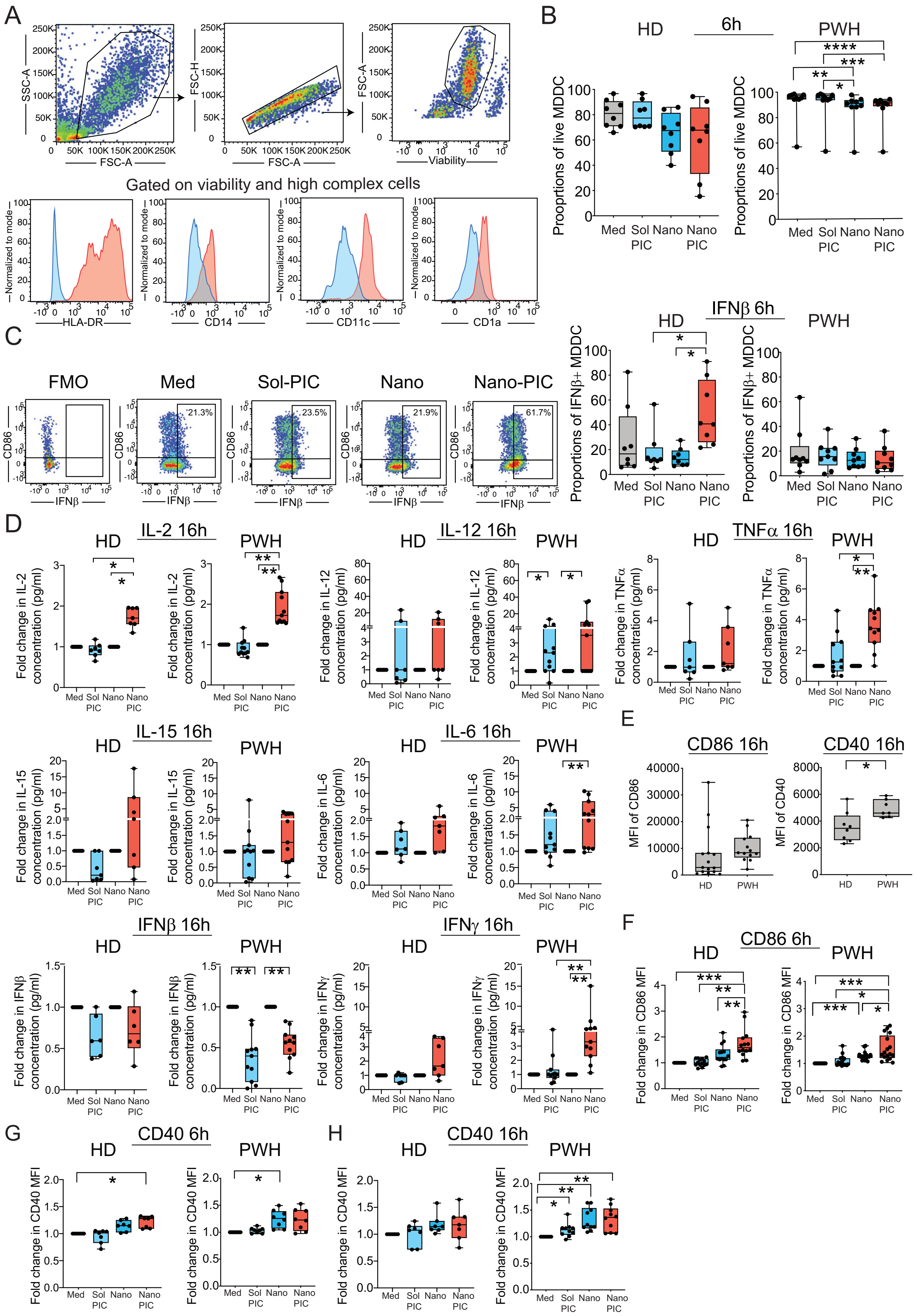
A
SSC-A
FSC-A
FSC-H
Viability
Gated on viability and high complex cells
Normalized to mode
HLA-DR
CD14
CD11c
CD1a
B
HD
6h
PWH
Proportions of live MDDC
Med Sol PIC Nano Nano PIC
C
FMO
Med
Sol-PIC
Nano
Nano-PIC
CD86
IFNβ
21.3%
23.5%
21.9%
61.7%
IFNβ 6h
Proportions of IFNβ+ MDDC
D
IL-2 16h
Fold change in IL-2 concentration (pg/ml)
IL-12 16h
Fold change in IL-12 concentration (pg/ml)
TNFα 16h
Fold change in TNFα concentration (pg/ml)
IL-15 16h
Fold change in IL-15 concentration (pg/ml)
IL-6 16h
Fold change in IL-6 concentration (pg/ml)
E
CD86 16h
MFI of CD86
CD40 16h
MFI of CD40
IFNβ 16h
Fold change in IFNβ concentration (pg/ml)
IFNγ 16h
Fold change in IFNγ concentration (pg/ml)
F
CD86 6h
Fold change in CD86 MFI
G
CD40 6h
Fold change in CD40 MFI
H
CD40 16h

 © The Author(s)

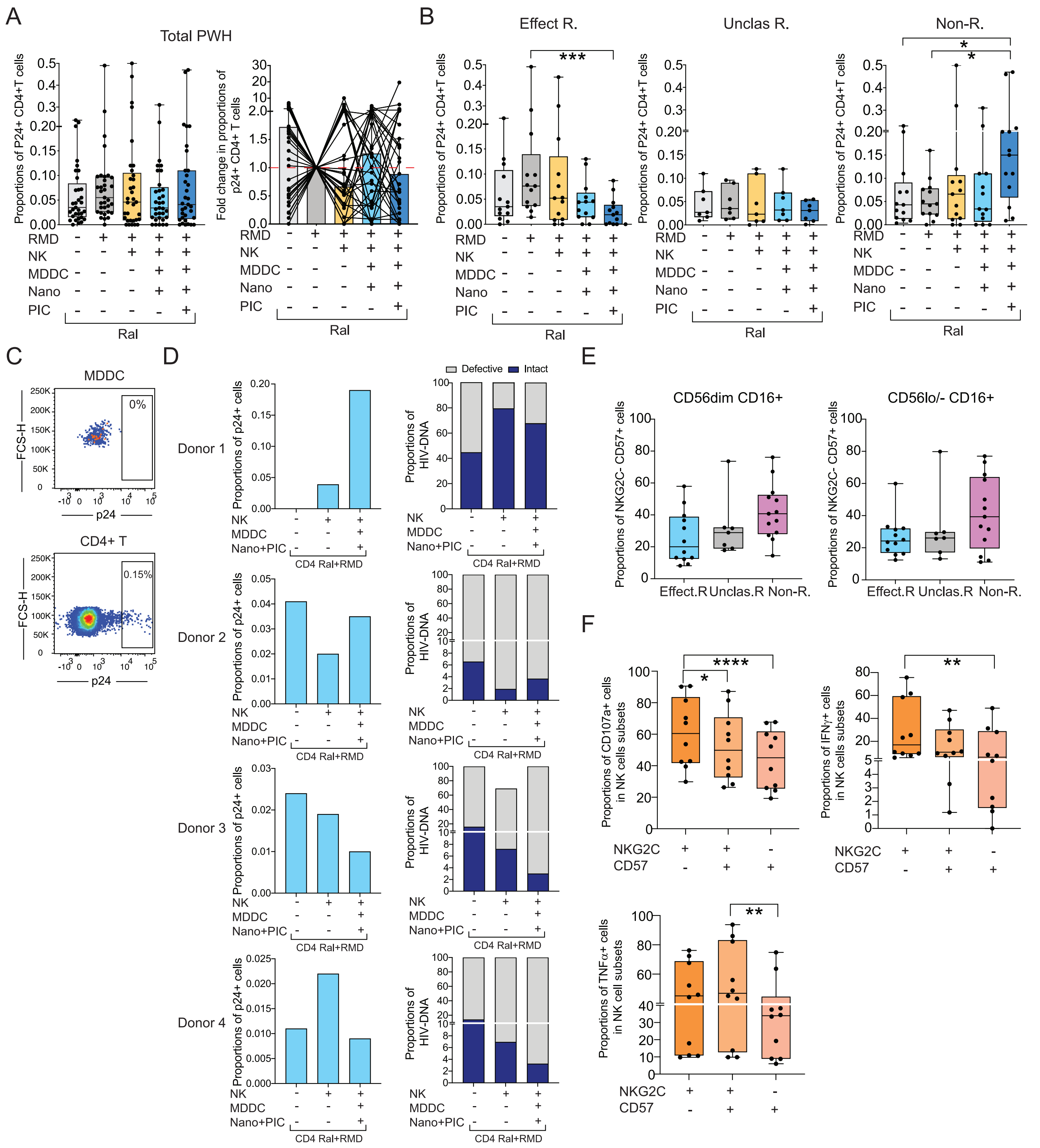
A
Total PWH
Proportions of P24+ CD4+T cells
Fold change in proportions of p24+ CD4+ T cells
RMD
NK
MDDC
Nano
PIC
Ral
B
Effect R.
Unclas R.
Non-R.
***
*
*
Proportions of P24+ CD4+T cells
C
MDDC
0%
FCS-H
p24
CD4+ T
0.15%
D
Donor 1
Donor 2
Donor 3
Donor 4
Proportions of p24+ cells
Proportions of HIV-DNA
Defective
Intact
NK
MDDC
Nano+PIC
CD4 Ral+RMD
E
CD56dim CD16+
CD56lo/- CD16+
Proportions of NKG2C- CD57+ cells
Effect.R
Unclas.R
Non-R.
F
Proportions of CD107a+ cells in NK cells subsets
Proportions of IFNγ+ cells in NK cells subsets
Proportions of TNFα+ cells in NK cell subsets
****
*
**
**
NKG2C
CD57

Figure EV2. Functional restoration of NK from PWH eliminating HIV-1-infected CD4+ T cells after treatment with Nano-PIC-MDDC.

(**A**) Raw data and fold change in proportions of HIV-1 p24+ CD4+ T cells from all $n = 33$ PWH recruited for the study cultured with Romidepsin and Raltegavir in the absence or the presence of autologous NK cells alone or stimulated with Nano-empty or Nano-PIC-MDDC. (**B**) Proportions of HIV-1 p24+ CD4+ T cells in the presence of NK cells stimulated with Nano-PIC-MDDC from three separate effective responder (Effect. R; $n = 13$), unclassified (Unclas. R. $n = 7$) and non-responder (Non-R.; $n = 13$) PWH groups. (**C**) Representative flow cytometry dot plots showing intracellular expression of p24 in Nano-PIC-MDDC (upper plot) or co-cultured autologous CD4 + T cells (lower plot) from a tested PWH. (**D**) Proportions of HIV-1 p24+ cells analyzed by FACS (left plots, light blue) and IPDA analysis of intact (dark blue) or defective (gray) HIV-DNA in CD4+ T cells from $n = 4$ PWH cultured with Romidepsin and Raltegravir in the absence or the presence of autologous NK cells alone or stimulated with Nano-PIC-MDDC. (**E**) Proportions of NKG2C- CD57+ subset on CD56dim or CD56lo/- CD16 + NK from effective responder ($n = 13$, Effect. R; blue), unclassified ($n = 7$, Unclas. R; gray) and non-responder ($n = 13$, Non-R.; pink) PWH after activation with Nano-PIC-MDDC. (**F**) Proportions of total CD107a+ (left), total FNγ+ (right) and total TNFα+ (below) included in gated memory NK precursors NKG2C + CD57−, memory differentiated NKG2C + CD57+ and effector NKG2C− CD57+ subsets from $n = 10$ selected effective responder PWH after Nano-PIC-MDDCs in presence of PMA and Ionomycin stimulation for 4 h and Brefeldin A and Monensin. Data from (**A**, **B**, **E**, **F**) are presented in Box and Whiskers plots showing median values and maximum and minimum error bars. The data in (**D**) are represented with bar plots. Statistical Significance was calculated using a Friedman or a Kruskal–Wallis test for multiple comparisons. *$P < 0.05$; **$P < 0.01$; ***$P < 0.001$.

© The Author(s)

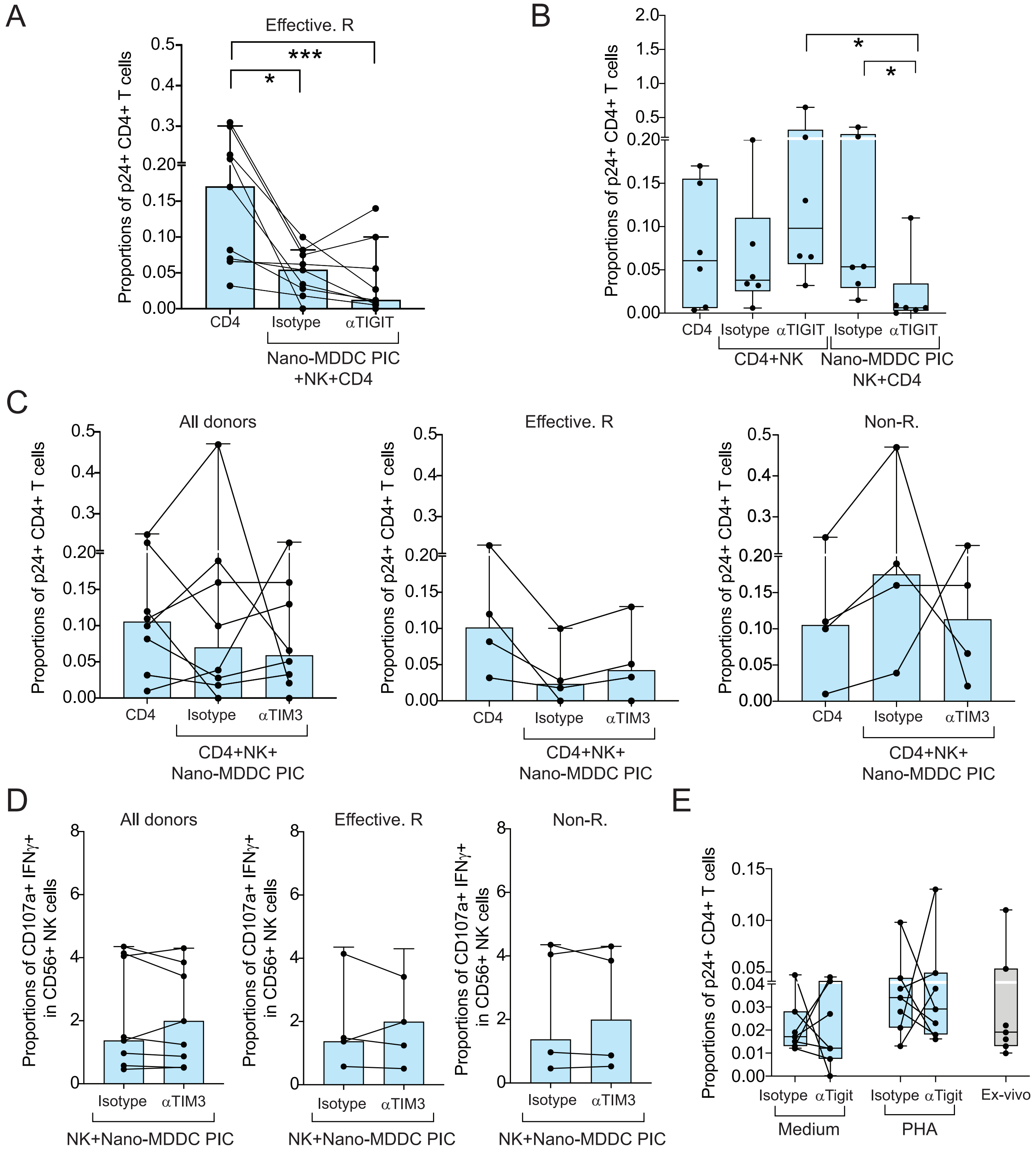
A
Effective. R
***
*
Proportions of p24+ CD4+ T cells
0.5
0.4
0.3
0.20
0.15
0.10
0.05
0.00
CD4
Isotype
αTIGIT
Nano-MDDC PIC
+NK+CD4
B
2.0
1.5
1.0
0.5
0.20
0.15
0.10
0.05
0.00
*
*
Proportions of p24+ CD4+ T cells
CD4
Isotype
αTIGIT
Isotype
αTIGIT
CD4+NK
Nano-MDDC PIC
NK+CD4
C
All donors
Effective. R
Non-R.
Proportions of p24+ CD4+ T cells
CD4
Isotype
αTIM3
CD4+NK+
Nano-MDDC PIC
D
All donors
Effective. R
Non-R.
Proportions of CD107a+ IFNγ+
in CD56+ NK cells
8
6
4
2
0
Isotype
αTIM3
NK+Nano-MDDC PIC
E
Proportions of p24+ CD4+ T cells
0.15
0.10
0.05
0.04
0.03
0.02
0.01
0.00
Isotype
αTigit
Isotype
αTigit
Ex-vivo
Medium
PHA

Figure EV3. Proportions of p24+ CD4+ T cells after TIGIT and TIM3 blocking antibody.

(**A**, **B**) Proportions of CD4+ T cells expressing HIV-1 p24+ cells cultured alone or with NK treated with Nano-PIC-MDDC from $n = 9$ effective responder PWH (**A**) or just media from $n = 6$ non-responder PWH (**B**) in the presence of isotypic control or anti-TIGIT mAbs. (**C**) Analysis of impact of blocking anti-TIM3 mAbs in experiments performed under the same conditions as (**A**) using $n = 8$ total PWH (left) or stratified based on effective ($n = 4$, middle) and non-responders ($n = 4$, right) donors. (**D**) Proportions of CD107a+ IFNγ + CD56 + NK cells in the co-culture experimental conditions described in (**C**) in the three responder PWH groups previously mentioned. (**E**) Proportions of p24+ cells in CD4+ T cells from $n = 7$ PWH treated with isotypic control or anti-TIGIT mAbs in the presence of media alone (basal) or under reactivation conditions with PHA (PHA). Baseline ex vivo levels of p24+ cells form these PWH are shown in the gray box and whisker bar. Data are presented in Box and Whiskers plots showing median values and maximum and minimum error bars. Statistically significant differences were calculated using a Friedman test for multiple comparisons and in the (**B**) was calculated using a one tailed Wilcoxon test and Bonferroni correction was applied. *$P < 0.05$; ***$P < 0.001$.

 © The Author(s)

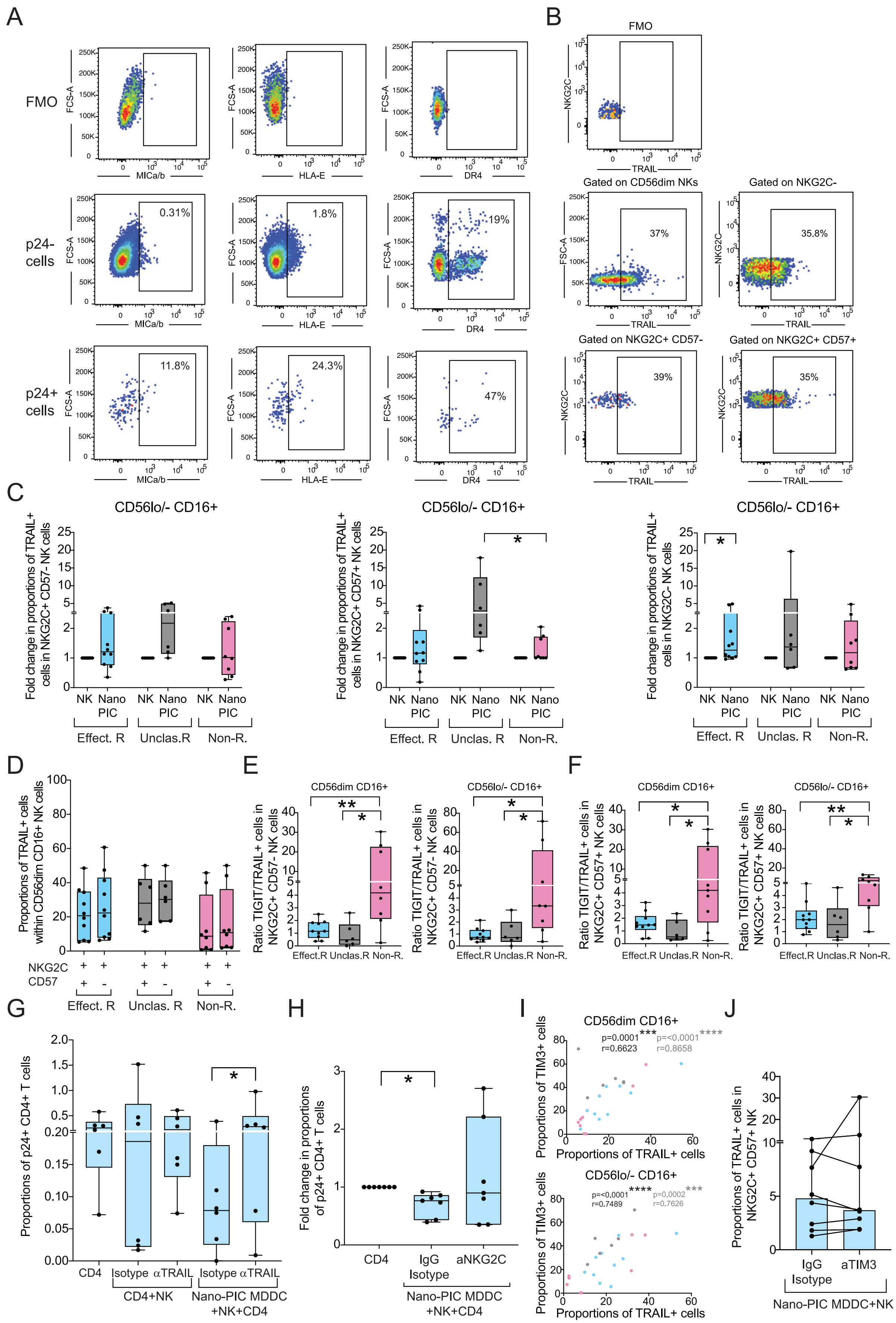
A
B
FMO
p24- cells
p24+ cells
MICa/b
HLA-E
DR4
FCS-A
0.31%
1.8%
19%
11.8%
24.3%
47%
TRAIL
NKG2C
Gated on CD56dim NKs
Gated on NKG2C-
Gated on NKG2C+ CD57-
Gated on NKG2C+ CD57+
37%
35.8%
39%
35%
C
CD56lo/- CD16+
Fold change in proportions of TRAIL+ cells in NKG2C+ CD57- NK cells
Fold change in proportions of TRAIL+ cells in NKG2C+ CD57+ NK cells
Fold change in proportions of TRAIL+ cells in NKG2C- NK cells
NK Nano PIC
Effect. R
Unclas.R
Non-R.
D
Proportions of TRAIL+ cells within CD56dim CD16+ NK cells
NKG2C
CD57
E
CD56dim CD16+
CD56lo/- CD16+
Ratio TIGIT/TRAIL+ cells in NKG2C+ CD57- NK cells
F
Ratio TIGIT/TRAIL+ cells in NKG2C+ CD57+ NK cells
Effect.R Unclas.R Non-R.
G
Proportions of p24+ CD4+ T cells
CD4
Isotype αTRAIL
CD4+NK
Nano-PIC MDDC +NK+CD4
H
Fold change in proportions of p24+ CD4+ T cells
IgG Isotype
aNKG2C
I
CD56dim CD16+
p=0.0001 r=0.6623
p=<0.0001 r=0.8658
Proportions of TIM3+ cells
Proportions of TRAIL+ cells
CD56lo/- CD16+
p=<0.0001 r=0.7489
p=0.0002 r=0.7626
J
Proportions of TRAIL+ cells in NKG2C+ CD57+ NK
aTIM3
Nano-PIC MDDC+NK

Figure EV4. Analysis of expression of NK receptor ligands on p24- vs p24+ CD4+ T cells and TRAIL on NK cell subsets from different PWH and its association with functionality.

(**A**) Representative flow cytometry dot plots for the NK receptor ligands MIC/ab (NKG2D), HLA-E (NKG2C/A) and DR4 (TRAIL), in gated p24+ or p24- CD4 + T cells from PWH after 16h culture with PHA + IL-2. A FMO control is included to each NK ligand. (**B**) Representative Flow cytometry dot plot showing expression of TRAIL on total CD56dim CD16+ NK, total NKG2C-, NKG2C+ CD57- and NKG2C+ CD57+ NK from a representative effective responder PWH. (**C**) Fold change in proportions of TRAIL+ cells in NKG2C- (left) and adaptive NKG2C+ CD57- (middle) and NKG2C+ CD57+ (right) subsets after Nano-PIC-MDDC from effective responder (*n* = 10; Effect. R; blue), unclassified (*n* = 6; Unclas. R; gray) and non-responder (*n* = 8; Non-R.; pink) PWH included on CD56lo/- CD16 + NK. (**D**) Raw proportions of TRAIL+ cells within adaptive NKG2C+ CD57+ and NKG2C+ CD57− cell subsets in the same responders PWH groups defined in (**C**) included in CD56dim CD16+ NK subpopulation after stimulation with Nano-PIC-MDDC. Statistical significance was calculated using a Mann–Whitney test and Bonferroni correction. (**E**, **F**) Ratio of TIGIT+ versus TRAIL+ cells in precursor NKG2C+ CD57- adaptive (**E**) and mature NKG2C + CD57+ (**F**) cells in CD56dim (left) and CD56lo/− (right) CD16 + NK subsets from the same PWH responder groups. Statistical significance was calculated using a one tale Mann–Whitney test and Bonferroni correction. (**G**, **H**) Proportions of HIV-p24 + CD4 + T cells from *n* = 6 (**G**) and *n* = 7 (**H**) PWH previously identified as effective responders treated with Raltegravir and Romidepsin and cultured in the absence or the presence of autologous unstimulated NK cells or NK treated with Nano-PIC-MDDCs in the presence of either IgG Isotypic control or with anti-TRAIL blocking mAb (**G**) or in the presence of anti-NKG2C mAb (**H**). Statistical significance was calculated using a one tale Wilcoxon matched pairs test and Bonferroni correction or a Friedman test for multiple comparisons. (**I**) Spearman correlations between proportions of TIM3 and TRAIL after Nano-PIC-MDDC within CD56dim (upper) and CD56lo/− (bottom) CD16 + NK. Statistical *P* and *R* values considering all data (black), without unclassified group (gray). (**J**) Analysis of proportions of TRAIL+ cells within adaptive NKG2C + CD57 + NK from *n* = 7 PWH stimulated with Nano-PIC-MDDC in the presence of isotype or anti-TIM3 mAb. Statistical significance was calculated were calculated using a two-tailed Wilcoxon matched pairs. Data from (**C–H**, **J**) are presented in Box and Whiskers plots showing median values and maximum and minimum error bars. *$P < 0.05$; **$P < 0.01$; ***$P < 0.001$; ****$P < 0.0001$.

© The Author(s)

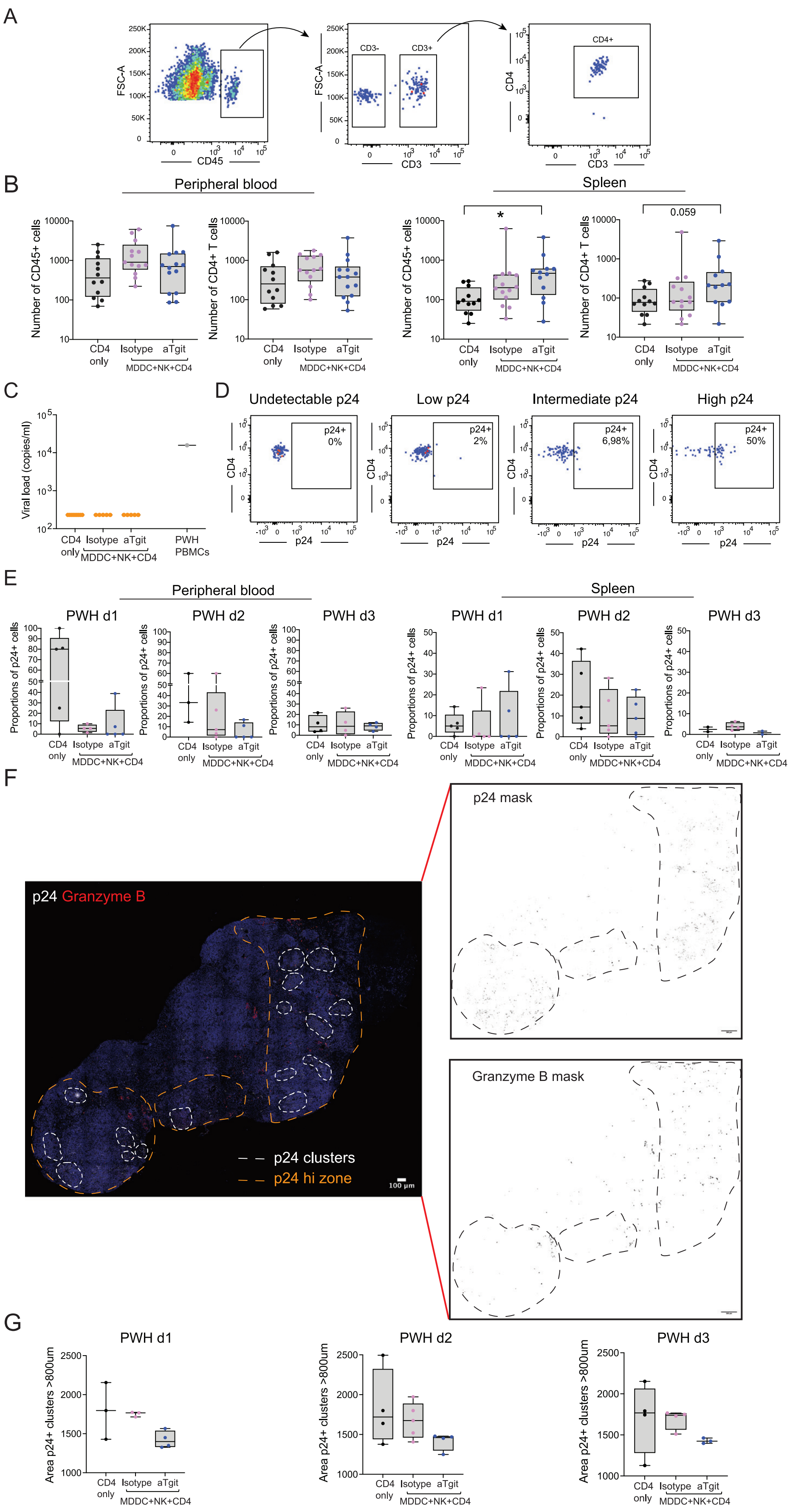
A
FSC-A
CD45
CD3-
CD3+
CD3
CD4
CD4+
B
Peripheral blood
Spleen
Number of CD45+ cells
Number of CD4+ T cells
*
0.059
CD4 only
Isotype
aTgit
MDDC+NK+CD4
C
Viral load (copies/ml)
PWH PBMCs
D
Undetectable p24
Low p24
Intermediate p24
High p24
p24+ 0%
p24+ 2%
p24+ 6,98%
p24+ 50%
p24
E
PWH d1
PWH d2
PWH d3
Proportions of p24+ cells
F
p24 Granzyme B
p24 clusters
p24 hi zone
100 µm
p24 mask
Granzyme B mask
G
Area p24+ clusters >800um

◀ **Figure EV5. Analysis of impact of anti-TIGIT antibodies on HIV-1 reactivation and in reconstitution and histological patterns in humanized NSG mice transplanted with NK cells.**

(**A**) Representative flow cytometry gating strategy showing identification of human CD45+ cells from PWH transplanted into immunodeficient NSG mice, and the identification of CD4+ CD3+ T cells. (**B**) Total absolute numbers of human CD45+ (left) and CD4+ T cells (right) in the peripheral blood and spleen from NSG mice transplanted either with CD4 + T cells from PWH alone (black) or in combination with autologous NK and Nano-PIC-MDDC and injected with either Isotypic (violet) or anti-TIGIT (blue). Data from $n = 3$ independent experiments using different PWH donors is shown. (**C**) Analysis of HIV-1 plasma viral load on plasma of transplanted NSG mice receiving either only CD4 + T cells from PWH or Nano-PIC-MDDC-NK immunotherapy and either Isotypic or anti-TIGIT mAbs. A positive control from cells from PWH is also shown. (**D**) Representative examples of levels of intracellular HIV-1 p24 on gated circulating human CD4 + CD3+ transplanted into mice and showing undetectable, low, intermediate and high p24 detection. (**E**) Proportions of HIV-1 p24+ cells within CD4 + T cells in the peripheral blood (left plot) and spleen (right plot) of NSG mice transplanted with CD4 + T cells from PWH alone (black) or in combination with autologous NK cells and Nano-PIC-MDDCs and injected with either Isotypic (violet) or anti-TIGIT (blue) mAbs for each donors 1, 2 and 3 (d1, d2, d3) used on each independent experiment. (**F**) Representative confocal microscopy images showing immunofluorescence histological analysis of a spleen tissue section from a representative transplanted NSG mouse showing staining of HIV-1 p24 (white) and granzyme B (red) showing areas with areas of high p24 cluster concentration (orange) and individual p24 clusters (white). Quantification of individual cluster cells with p24 (upper right) and granzyme B (lower right) staining masks are shown. Scale bar: 100 µm. (**G**) Analysis of size of clusters of p24+ cells present in the spleen of NSG mice transplanted with the three different d1, d2, d3 PWH as previously described in (**E**). In panel (**B**, **E**, **G**) data are presented in Box and Whiskers plots showing median values and maximum and minimum error bars. Statistical significant differences between the different groups of treatment were calculated using a two tail Mann–Whitney test and Bonferroni correction for multiple comparisons. $^*P < 0.05$.

 © The Author(s)
